# Transthoracic ultrasound planning in the treatment of second and third stage of empyema

**DOI:** 10.1186/1749-8090-8-S1-O71

**Published:** 2013-09-11

**Authors:** N Asadi, G Caroli, G Dolci, A Dell'Amore, D Greco, C Ammari, A Bini, F Stella

**Affiliations:** 1 General Thoracic Surgery, Cardiothoracic Department, S.Orsola-Malpighi University Hospital, Bologna, Italy

## Background

The management of empyema is still debated. Video-assisted thoracoscopic surgery (VATS) has revolutionized surgical management of patients with empyema. Currently thoracoscopic approach probably is the best choice in the management of second or third stage of empyema, particularly in those patients with chronic empyema and poor performance status. Multioculated empyemas generally make thoracoscopic procedure difficult therefore open procedures would be preferable. We evaluate the effectiveness of transthoracic ultrasound (TUS) in the planning of the best thoracoscopic approach in treatment of chronic empyema.

## Methods

From January 2007 to March 2013 28 patients with pleural empyema underwent preoperative transthoracic ultrasound. II stage of empyema was presented in 23 patients.

Patients were examined in surgical position. TUS was used to analyze and localize multi loculations pockets of pleural effusion and pneumonia with consolidation, the hemidiaphragm and the major pockets of the pleural effusion were marked on the skin. In consideration of the TUS and the signs, a small working window and a thoracoscopic access were drawn on the skin of the patient. TUS accuracy was determinate on empyema staging, topography of multilocalations pleural effusion, hemidiaphragm localization and finally thoracoscopic accesses pianification.

## Results

All patients underwent thoracoscopic treatment with a working window of about 10 cm and a second access of 10mm. In 4 cases (14,2%) open-convertion was needed for pathology extention. Topography was correct in 26 cases (92,8%) and good thoracoscopic approach was achieved in 24 (85,7%). The presence of small and multiple loculations and a hyperechoic effusion were the main causes of failure.

## Conclusions

TUS is a very useful method in the planning of thoracoscopic treatment of pleural empyema. The utility of this method should be further extended in the treatment of this pathology.

